# Replication-stress-associated DSBs induced by ionizing radiation risk genomic destabilization and associated clonal evolution

**DOI:** 10.1016/j.isci.2021.102313

**Published:** 2021-03-15

**Authors:** Yusuke Matsuno, Mai Hyodo, Mafuka Suzuki, Yosuke Tanaka, Yasunori Horikoshi, Yasufumi Murakami, Hidetaka Torigoe, Hiroyuki Mano, Satoshi Tashiro, Ken-ichi Yoshioka

**Affiliations:** 1 Laboratory of Genome Stability Maintenance, National Cancer Center Research Institute, Tsukiji, Chuo-ku, Tokyo 104-0045, Japan; 2 Department of Applied Chemistry, Faculty of Science, Tokyo University of Science, Kagurazaka, Shinjuku-ku, Tokyo 162-8601, Japan; 3 Biological Science and Technology, Tokyo University of Science, Niijuku, Katsushika-ku, Tokyo 125-8585, Japan; 4 Division of Cellular Signaling, National Cancer Center Research Institute, Tsukiji, Chuo-ku, Tokyo 104-0045, Japan; 5 Department of Cellular Biology, Research Institute for Radiation Biology and Medicine, Hiroshima University, Hiroshima, Japan

**Keywords:** Biological Sciences, Cancer, Cell Biology

## Abstract

Exposure to ionizing radiation is associated with cancer risk. Although multiple types of DNA damage are caused by radiation, it remains unknown how this damage is associated with cancer risk. Here, we show that after repair of double-strand breaks (DSBs) directly caused by radiation (dir-DSBs), irradiated cells enter a state at higher risk of genomic destabilization due to accumulation of replication-stress-associated DSBs (rs-DSBs), ultimately resulting in clonal evolution of cells with abrogated defense systems. These effects were observed over broad ranges of radiation doses (0.25–2 Gy) and dose rates (1.39–909 mGy/min), but not upon high-dose irradiation, which caused permanent cell-cycle arrest. The resultant genomic destabilization also increased the risk of induction of single-nucleotide variants (SNVs), including radiation-associated SNVs, as well as structural alterations in chromosomes. Thus, the radiation-associated risk can be attributed to rs-DSB accumulation and resultant genomic destabilization.

## Highlights

• Replication-stress-associated DSBs accumulate after exposure to ionizing radiation
• Such DSBs risk genomic destabilization and associated mutagenesis
• The resulting genomic rearrangements and mutations lead to clonal evolution
• The radiation-associated risks arise at wide ranges of radiation doses and dose rates

## Graphical abstract

**Figure:**
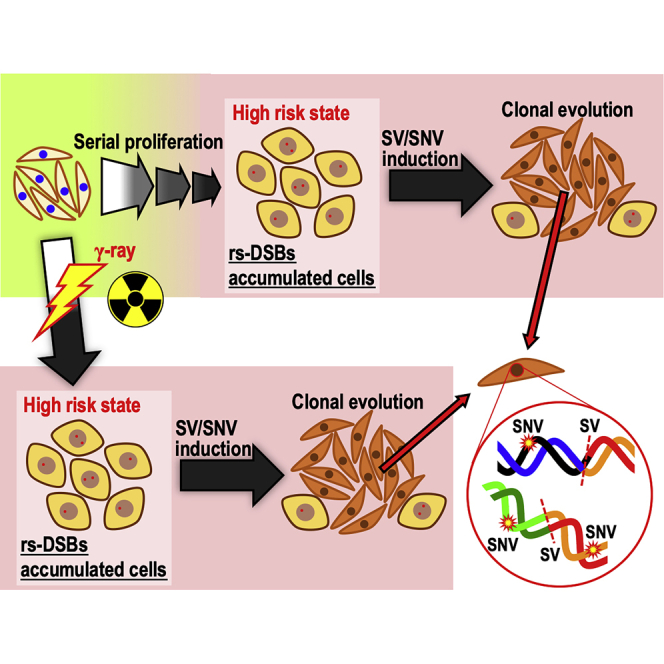

## Introduction

Radiation exposure is associated with cancer risk (Gilbert, 2009; Howe et al., 2006; Morgan and Sowa, 2015). Irradiation with X-rays or γ-rays causes DNA damage, including dir-DSBs and single-strand breaks (SSBs), as well as reactive oxygen species (ROS), which produce oxidized nucleotide adducts such as 8-oxoguanine (Borrego-Soto et al., 2015; Lomax et al., 2013; Suzuki et al., 2003). SSBs and ROS are also associated with rs-DSB induction (Burhans and Weinberger, 2007; Zeman and Cimprich, 2014). In addition, these are usually induced as clustered lesions (Georgakilas et al., 2013; Nickoloff et al., 2020; Sage and Shikazono, 2017). However, it remains unclear which types of damage, stress, and/or adducts caused by radiation are associated with cancer risk and how they lead to induction of mutation in cancer-driver genes. Given that radiation-associated cancers usually exhibit genomic instability (Burtt et al., 2016; Suzuki et al., 2003), it is possible that cancer risk is mediated by genomic destabilization and the associated induction of mutation in cancer-driver genes (Matsuno et al., 2019). However, it remains unclear how genomic destabilization risk is increased by irradiation.

Among the multiple types of damage, stress, and adducts caused by radiation, the best characterized are those caused by the cellular responses to dir-DSBs (Borrego-Soto et al., 2015; Lomax et al., 2013; Suzuki et al., 2003). These include immediate formation of γH2AX foci and the associated activation of checkpoint responses that induce cell-cycle arrest, repair, cellular senescence, and apoptosis (Mirzayans et al., 2012; Shiloh and Ziv, 2013). Although the level of H2AX, which mediates those responses, is generally reduced after the cellular growth rate is slowed (Yoshioka et al., 2012), dir-DSBs are still effectively repaired even in the H2AX-diminished state because H2AX is transiently upregulated under the control of ATM and SIRT6 (Atsumi et al., 2015). However, cells with repaired dir-DSBs subsequently accumulate persistent DSBs during subsequent replication (Minakawa et al., 2016). Given that rs-DSBs can trigger genomic destabilization (Matsuno et al., 2019), the DSBs arising during the subsequent replication in irradiated cells might be responsible for genomic destabilization and the associated clonal evolution of cells with abrogated defense systems.

In this study, we found that radiation risk is primarily associated with induction of a cellular state at higher risk of genomic destabilization and associated mutagenesis and showed that this risk is elevated due to accumulation of rs-DSBs. Ultimately, this can lead to clonal evolution of cells with abrogated defense systems.

## Results

### Induction of genomic instability in γ-irradiated cells

**Figure 1:**
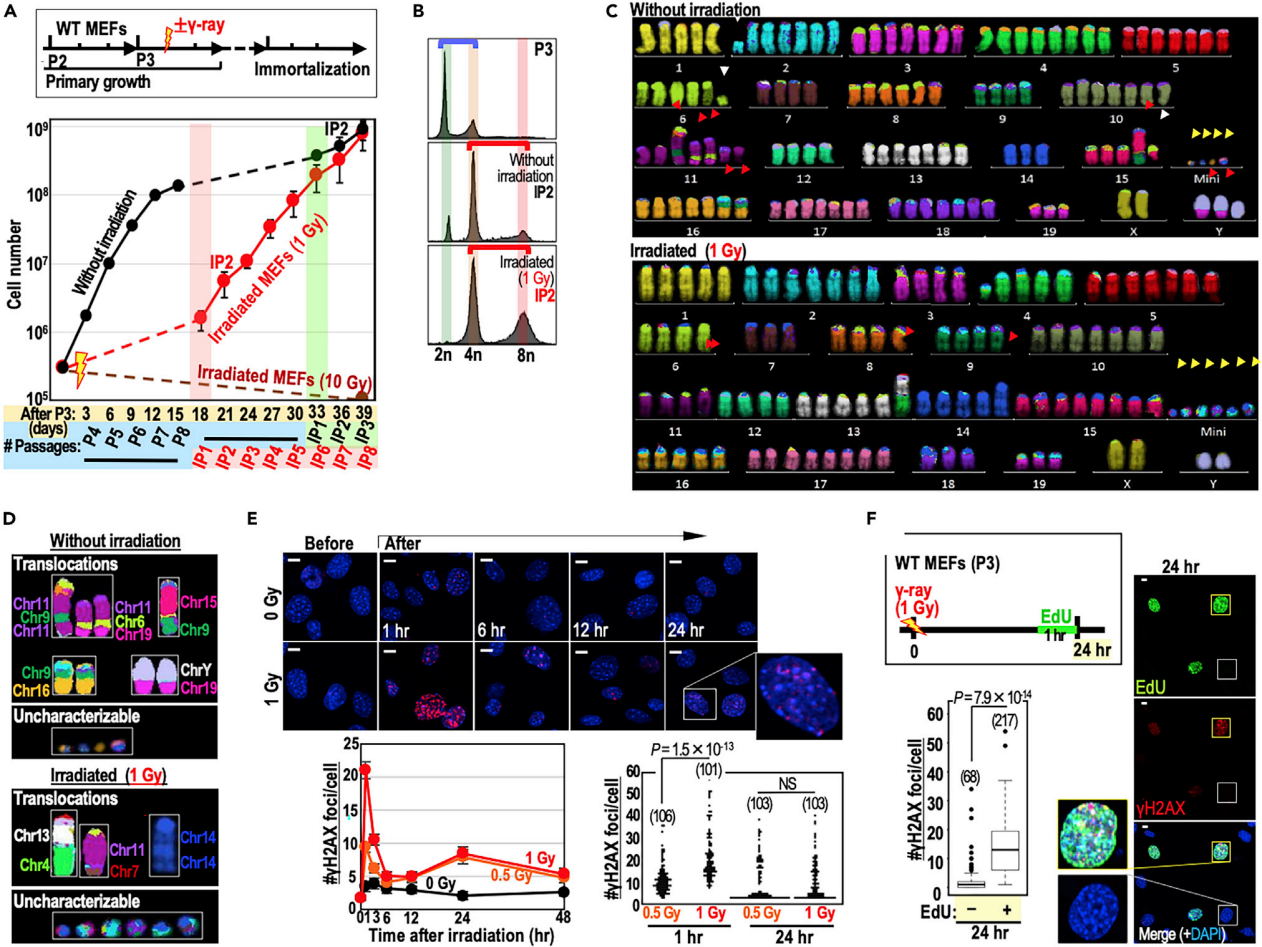
MEFs irradiated with 1 Gy γ-rays enter a state at higher risk of genomic destabilization, concomitant with accumulation of rs-DSBs (A) WT MEFs were irradiated with γ-rays and cultivated under the Std-3T3 protocol to monitor the immortalization process. The graph shows mean cell numbers ±s.d. (n = 3 independent experiments with MEFs prepared from independent fetuses). (B–D) CIN status was determined by flow cytometry (B) and multi-color FISH analyses (C and D). Blue and red bars in (B) indicate diploidy- and tetraploidy-associated peaks, respectively. Translocations, deletions, and origin-unclear mini-chromosomes are indicated by red, white, and yellow arrowheads, respectively (C). (E) WT MEFs were irradiated by γ-rays, and γH2AX focus status was monitored until 48 h after irradiation. Representative images are provided. Scale bars in images, 10 μm. Bars in the bottom left graph show means ± s.d. Bottom right: numbers of γH2AX foci 1 and 24 h after irradiation (0.5 and 1 Gy γ-ray). Two-tailed Welch's t test was used for statistical analysis. NS, not significant. (F) Experiments were performed as shown in the workflow. EdU-positive cells were co-immunostained for γH2AX. Representative images are provided. Scale bars, 10 μm. Box plots show median, third, and first quartiles; whiskers (median ±1.5 times interquartile range); and outliers. Two-tailed Welch's t test was used for statistical analysis. NS, not significant. See also Figure S1.

To examine the effects of γ-rays on genomic instability and cancer-driver mutations, we monitored the immortalization process of wild-type mouse embryonic fibroblast cells (WT MEFs) after irradiation (1 or 10 Gy at 909 mGy/min) at early passage (P3) because these cells usually immortalize with chromosomal instability (CIN) (Atsumi et al., 2011; Ichijima et al., 2010) and mutations in the ARF/p53 pathway (Matheu et al., 2007; Osawa et al., 2013). MEFs irradiated at either dose decreased their growth rates (Figure 1A) but to different degrees: MEFs irradiated with 10 Gy did not subsequently recover growth, whereas MEFs irradiated with 1 Gy subsequently immortalized with tetraploidy (Figures 1A and 1B). This immortalization occurred about 2 weeks faster than in non-irradiated controls. Given that WT MEFs usually immortalize after induction of CIN (tetraploidy) (Atsumi et al., 2011; Ichijima et al., 2010) and subsequent mutations in the ARF/p53 pathway (Matheu et al., 2007; Osawa et al., 2013), these results imply that irradiation facilitated induction of CIN.

Next, we analyzed the CIN status of immortalized MEFs by examining M-phase chromosome profiles, comparing the irradiated and non-irradiated backgrounds (Figures 1C, 1D, and S1). Multi-color FISH data revealed chromosome number alterations, chromosomal deletions and translocations, as well as mini-chromosome formation of uncertain origin in both types of immortalized MEFs (Figures 1C and 1D). Consistent with the induction of tetraploidy (Figure 1B), both types of immortalized MEFs had a chromosome number peak around 80 (Figure S1A). Supporting the idea of immortalization associated with clonal evolution, identical chromosomal abnormalities were observed in all six independent images of non-irradiated MEFs and three out of four independent images of 1 Gy-irradiated MEFs (Figure S1D; see fusions of Chr. 11, 6, and 19, and truncation of Chr. 4, respectively). The overall level of abnormalities was not increased by irradiation (Figures S1A and S1C). Thus, 1 Gy irradiation facilitated genomic destabilization and associated clonal evolution of immortalized cells, but this did not appear to be directly associated with the increase in genomic alterations.

### Secondary DSB formation in S phase after irradiation

A previous study reported formation of secondary DSBs after the repair of primary DSBs (Minakawa et al., 2016). Hence, we next monitored the levels of γH2AX foci (a damage marker) for 48 h after irradiation (0.5 and 1 Gy). As expected, after decay of the initial γH2AX foci within a few hours, secondary γH2AX foci were induced by 24 h after irradiation and persisted until 48 h (Figure 1E). The secondary foci were specifically induced in EdU-positive cells following pulse-treatment with EdU (Figure 1F), indicating that DSB formation occurred during replication, as expected for rs-DSBs. Given that rs-DSBs can trigger genomic destabilization (Matsuno et al., 2019), these breaks represent a major radiation-associated risk factor for genomic destabilization and resultant mutagenesis. Intriguingly, in contrast to the initial γH2AX levels, secondary γH2AX levels were identical between 0.5 Gy- and 1 Gy-irradiated backgrounds (Figure 1E), implying that the secondary damage effect could be caused by a wide range of radiation doses.

### Induction of genomic instability by a wide range of γ-ray doses

**Figure 2:**
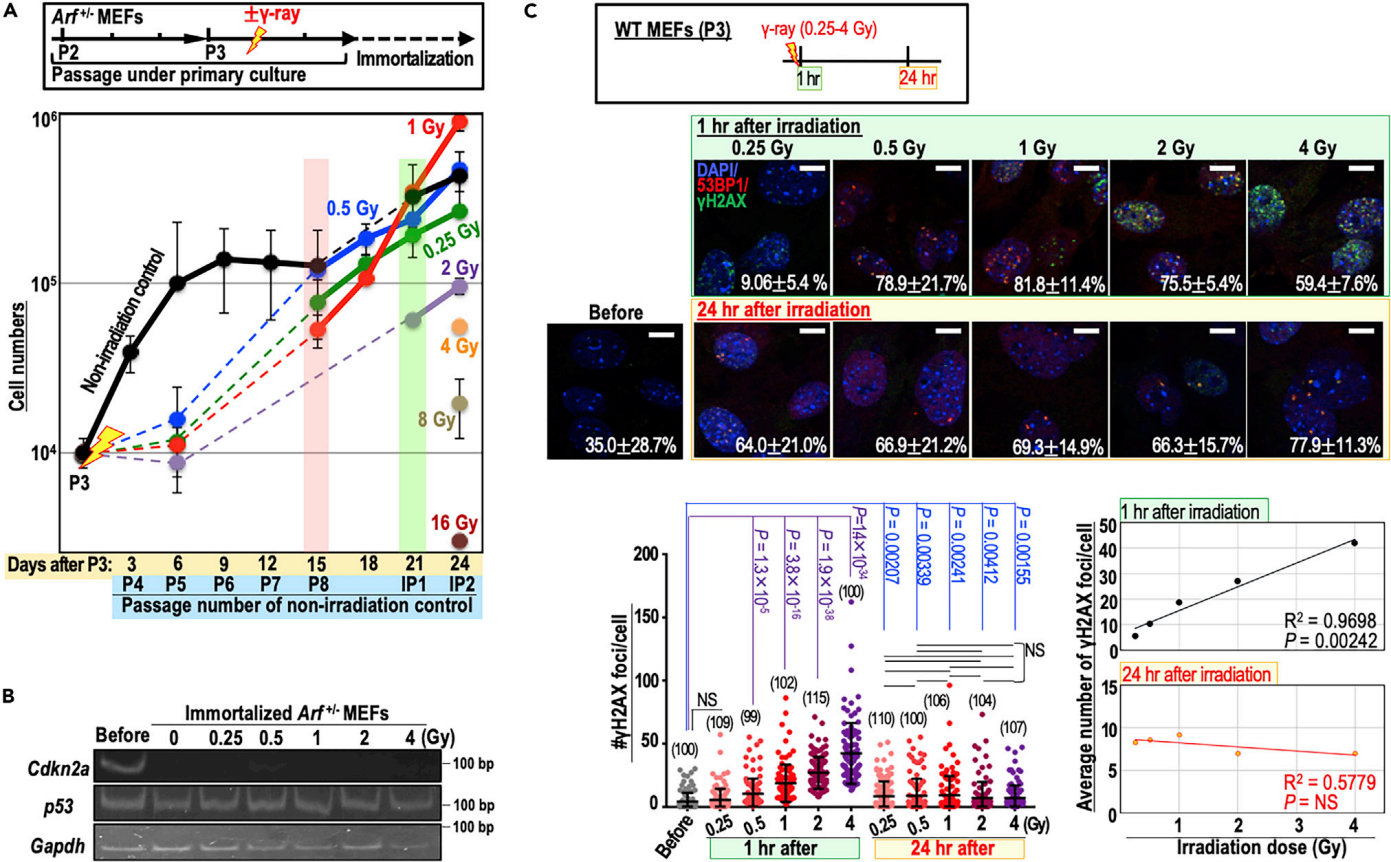
MEFs irradiated with a dose of 0.25–2 Gy undergo facilitated induction of genomic destabilization and loss of the *Cdkn2a*locus (A) *Arf*^+/−^ MEFs were irradiated with γ-rays (0.25–16 Gy) and cultivated under the Std-3T3 protocol to monitor the immortalization process. The graph shows mean cell numbers ±s.d. (n = 3 independent experiments with MEFs prepared from independent embryos). (B) Mutation induction status of the ARF/p53 module in *Arf*^+/−^ MEFs was monitored along with loss of the *Cdkn2a* locus, which encodes ARF. *p53* and *Gapdh* were used as internal controls. (C) *Arf*^+/−^ MEFs were irradiated with γ-rays, and γH2AX/53BP1 foci were analyzed 1 and 24 h after irradiation. Representative images are provided. Scale bars in images, 10 μm. Bars show means ± s.d. Correlation between average γH2AX-focus number and irradiated dose was evaluated 1 and 24 h after irradiation (right panels). Two-tailed Welch's t test was used for statistical analysis of left panels. Student's t test was used for statistical analysis of right panels. NS, not significant. See also Figure S2.

To test the effects of irradiation doses on the risk of genomic destabilization and the associated clonal evolution of mutated cells, we analyzed the immortalization process of *Arf*^+/−^ MEFs after irradiation (0.25–16 Gy at 909 mGy/min), as *Arf*^+/−^ MEFs usually immortalize with CIN and the resultant loss of the *Cdkn2a* locus (encoding ARF) (Osawa et al., 2013). Immortalization was induced in MEFs irradiated with 0.25–4 Gy γ-ray (Figure 2A), with concomitant loss of the *Cdkn2a* locus (Figure 2B) and CIN (Figures S2A and S2B). MEFs irradiated with 0.25–2 Gy γ-ray immortalized faster than non-irradiated controls (Figure S2C), indicating that irradiation facilitated induction of genomic destabilization. Such acceleration of genomic destabilization and resulting immortalization was observed over a wide dose range (0.25–2 Gy), which was much lower than the dose range that induced cell death (Figure S2D). Similar to the results shown in Figure 1D, chromosomal status was not significantly affected by irradiation dose as judged by numerical distributions (Figure S2B), implying that facilitation of genomic destabilization by radiation might be an indirect effect.

To follow the immortalization process of the irradiated *Arf*^+/−^ MEFs (P3), we performed time-lapse imaging and compared cells irradiated with 1 Gy with those treated with 10 Gy, as well as with non-irradiated controls (Videos S1, S2, and S3). Irradiated cells did not exhibit any visible change within the first few hours, but eventually slowed their growth and exhibited a flattened and enlarged morphology by 24 h, at a time when secondary γH2AX foci had accumulated (Figure 1E). After 24 h, MEFs irradiated with 1 Gy slowly proliferated and eventually developed actively proliferating fractions that formed colonies by day 5 (Figure S2E: see cells marked by dashed yellow line). This observation implies that genomically destabilized and mutated MEFs developed at least a few days prior to colony formation, i.e., 24–72 h after irradiation, at a time when cells had accumulated secondary γH2AX foci. Given that the phenotypes observed at 24–72 h after irradiation are generally seen when genomic destabilization risk is high (Yoshioka et al., 2012), the primary radiation risk is probably associated with genomic destabilization.

Video S1. *Arf*^+/−^ MEFs (P3) irradiated with 1 Gy γ-rays, related to Figure 2

Video S2. *Arf*^+/−^ MEFs (P3) irradiated with 10 Gy γ-rays, related to Figure 2

Video S3. *Arf*^+/−^ MEFs (P3) without irradiation, related to Figure 2

To test the statuses of initial and secondary DSBs, we monitored γH2AX/53BP1-focus levels 1 and 24 h after irradiation with 0.25–4 Gy. The number of γH2AX/53BP1 foci 1 h after irradiation increased in a dose-dependent manner, whereas the number of foci 24 h after irradiation was almost identical under all immortalized conditions (Figures 2C and S2F). This supports the idea of secondary DSB-dependent genomic destabilization over a wide range of radiation doses. Those effects were observed even under conditions in which dir-DSBs were rare (Figure 2C: see 1 h after 0.25 Gy irradiation), supporting the idea that dir-DSBs are separate from induction of CIN.

### Induction of genomic instability by a wide range of γ-ray dose rates

**Figure 3:**
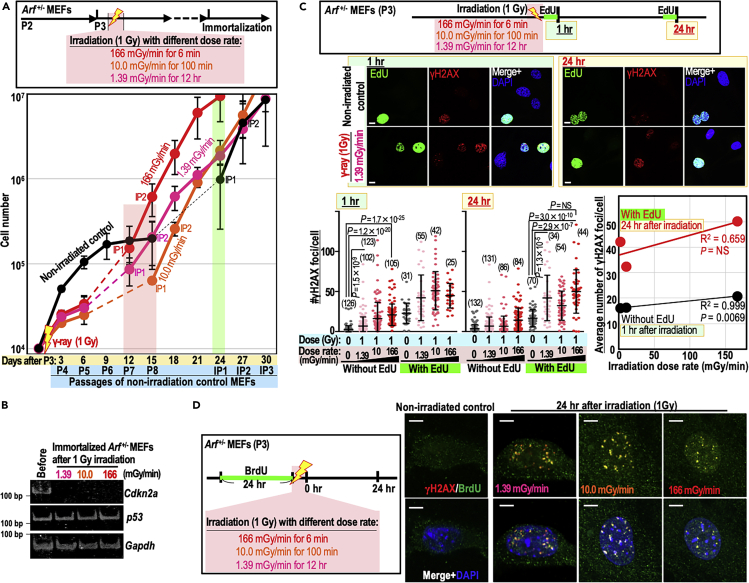
rs-DSBs arising due to irradiation at a wide range of dose rates cause identical genomic destabilization and mutation induction in the ARF/p53 module (A) *Arf*^+/−^ MEFs were irradiated with 1 Gy γ-rays at the dose rates shown in the workflow and cultivated under the Std-3T3 protocol to monitor the immortalization process. The graph shows mean cell numbers ±s.d. (n = 3 independent experiments with MEFs prepared from independent fetuses). (B) Mutation induction status of the ARF/p53 module: *Arf*^+/−^ MEFs were monitored for loss of the *Cdkn2a* gene, which encodes ARF. (C) *Arf*^+/−^ MEFs were treated as shown in the workflow. EdU-positive MEFs were co-immunostained with γH2AX. Representative images are provided. Bars in bottom left and middle panels show means ± s.d. Scale bars in images, 10 μm. Correlation between average γH2AX-focus number and irradiated dose rate was evaluated 1 h after irradiation in EdU-negative MEFs and 24 h after irradiation in EdU-positive MEFs (bottom right panel). Two-tailed Welch's t test was used for statistical analysis of left panels. Student's t test was used for statistical analysis of right panels. NS, not significant. (D) *Arf*^+/−^ MEFs were treated as shown in the workflow. Foci containing γH2AX and BrdU were detected by immunofluorescence under native conditions. Scale bars, 10 μm. See also Figure S3.

Biological responses to radiation could be altered by differences in irradiation dose rates, even when the total irradiation dose is the same. To test the effects of irradiation dose rate, we irradiated *Arf*^+/−^ MEFs 1 Gy at 1.39, 10.0, or 166 mGy/min. As with MEFs irradiated at 909 mGy/min (Figure 2), we observed facilitated immortalization under all conditions (Figure 3A), in association with loss of the *Cdkn2a* locus (Figure 3B) and genomic instability (Figure S3A). These observations indicate that genomic destabilization and the resultant *Cdkn2a* mutations are inducible over a wide range of radiation dose rates (1.39–909 mGy/min).

To analyze the damage status of MEFs irradiated at lower dose rates, we monitored γH2AX foci after treating *Arf*^+/−^ MEFs according to the workflow shown in Figure 3C. We specifically compared γH2AX foci levels 1 h after irradiation in EdU-negative MEFs, mainly reflecting dir-DSBs, and 24 h after irradiation in EdU-positive MEFs, mainly reflecting rs-DSBs. As expected, although the former varied with dose rate, the latter arose under all conditions tested regardless of dose rate (Figures 3C and S3B), supporting the correlation of secondary γH2AX formation with facilitated CIN induction.

Loci under replication stress expose ssDNA and hence are detectable under native conditions as foci of pre-incorporated BrdU (Toledo et al., 2013). To confirm those secondary foci as rs-DSBs, we tested the status of native BrdU foci co-localized with γH2AX and 53BP1 after the cells were treated according to the workflow shown in Figure 3D. As expected, γH2AX and 53BP1 foci arising 24–48 h after irradiation were mostly co-localized with native BrdU foci under all dose-rate conditions (Figures 3D and S3C). Therefore, we concluded that those secondary foci represented rs-DSBs. Together, our results showed that rs-DSBs are caused by a wide range of radiation doses (0.25–2 Gy) and dose rates (at least 1.39–909 mGy/min), ultimately risking genomic destabilization and mutation induction in the ARF/p53 module.

### Irradiation independence in genomic alteration rates

**Figure 4:**
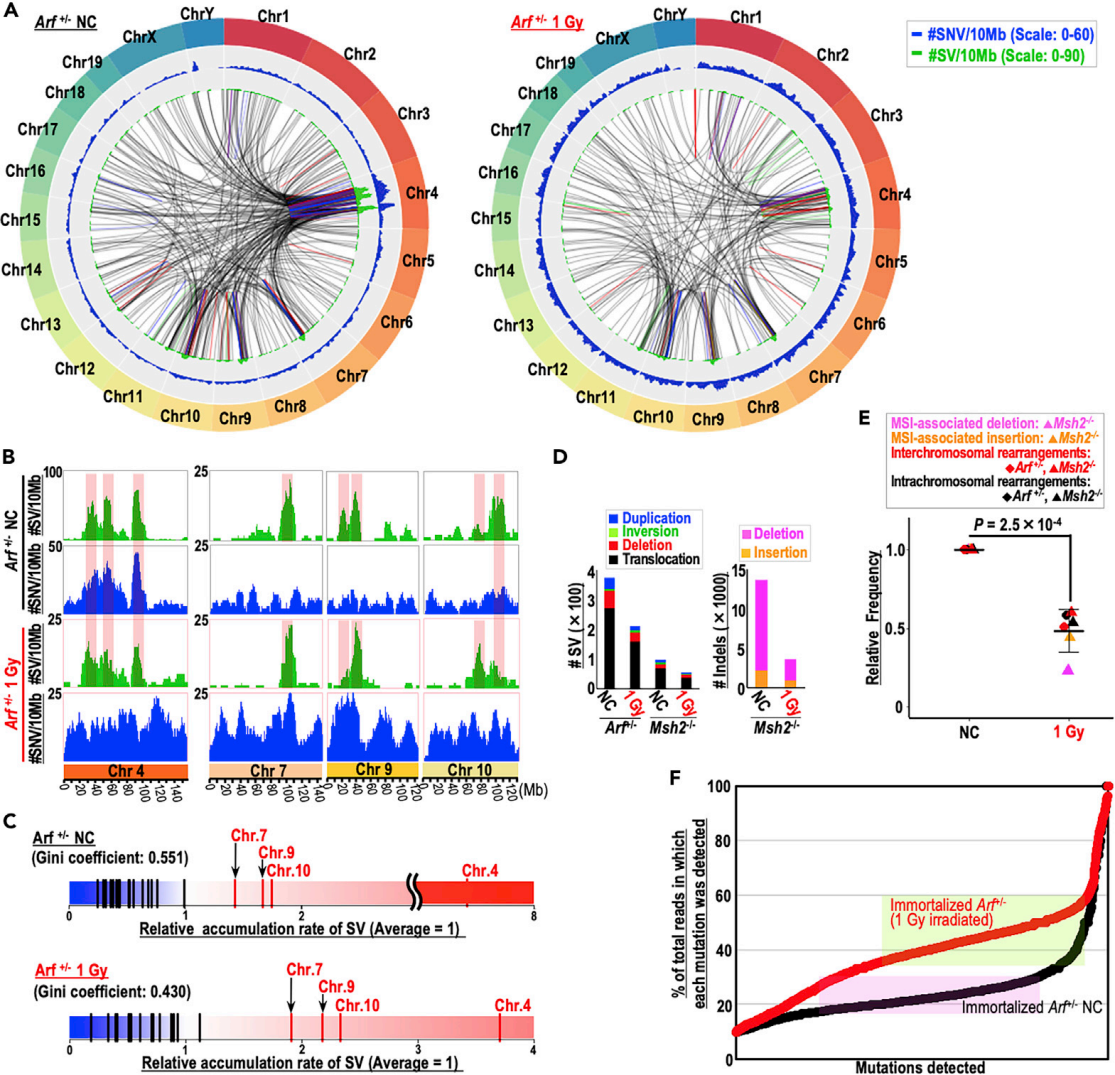
Massive SV is induced in a biased fashion in immortalized *Arf*^+/−^ MEFs in both irradiated and non-irradiated backgrounds (A) Genome-wide Circos plots of SVs and SNVs are shown. Chromosome ideograms are shown around the outer ring. The two inner circular tracks show numbers of SVs (green) and SNVs (blue) with the corresponding moving averages. Inside lines indicate duplications (blue lines), inversions (green lines), deletions (red lines), and translocations (black lines). (B) Numbers of SVs (green) and SNVs (blue) in chromosomes 4, 7, 9, and 10 are indicated with the corresponding moving averages. Peaks observed for MEFs in both non-irradiated and 1 Gy-irradiated backgrounds are marked with pink lines. (C) Relative accumulation rates of SV in each chromosome are indicated. (D) Numbers of each type of SV are shown in the left panel. Numbers of indels reflecting MSI in *Msh2*^−/−^ MEFs are shown in the right panel. (E) Relative rates of each type of genomic alteration. Two-tailed Welch's t test was used for statistical analysis. (F) Mutations detected in immortalized *Arf*^+/−^ MEFs. Percentage of reads detected as mutations is shown. See also Figure S4.

To investigate the effects of irradiation on genomic alterations, we analyzed whole-genome sequences of immortalized *Arf*^+/−^ MEFs and mismatch repair (MMR)-deficient *Msh2*^−/−^ MEFs and compared 1 Gy-irradiated and non-irradiated backgrounds; MEFs in primary growth (P3) were used as controls. In immortalized *Arf*^+/−^ MEFs, massive structural variants (SVs) were induced in a biased manner in specific loci (e.g., chromosome 4) (Figures 4A–4C), in contrast to the situation in *Msh2*^−/−^ MEFs (Figures S4A and S4B). Irradiation basically did not affect SV induction hotspots in *Arf*^+/−^ MEFs (Figures 4A–4C). Overall, SV numbers were decreased by irradiation (Figure 4D). Similarly, in *Msh2*^−/−^ MEFs that exhibited fewer SVs and massive insertions/deletions (Indel), as expected in MMR-deficient cells, the SV and indel numbers were also reduced under the irradiated background (Figures 4D and 4E). These observations are consistent with the results shown in Figures 1, 2, and 3: although γ-ray irradiation leads to facilitated genomic destabilization, this is not due to direct induction of genomic alteration.

More than 50% of SNVs were detected as mutations in 35%–60% of total reads in irradiated *Arf*^+/−^ diploid MEFs and in 18%–30% of total reads in non-irradiated *Arf*^+/−^ MEFs exhibiting tetraploidy (Figures 4F and S4C). These observations indicate that the resultant MEFs were a clone of a single MEF in which the majority of mutations were induced in a single chromosomal allele. Given that the immortalized *Arf*^+/−^ MEFs lost *Cdkn2a* (Figure 2B), such a process would be associated with the clonal evolution of cells mutated in ARF. *Msh2*^−/−^ MEFs were also immortalized through clonal evolution (Figure S4D). Together, our results revealed that irradiation leads to a state at higher risk of genomic destabilization and associated mutagenesis, ultimately risking clonal evolution.

### Mutations in irradiated cells

**Figure 5:**
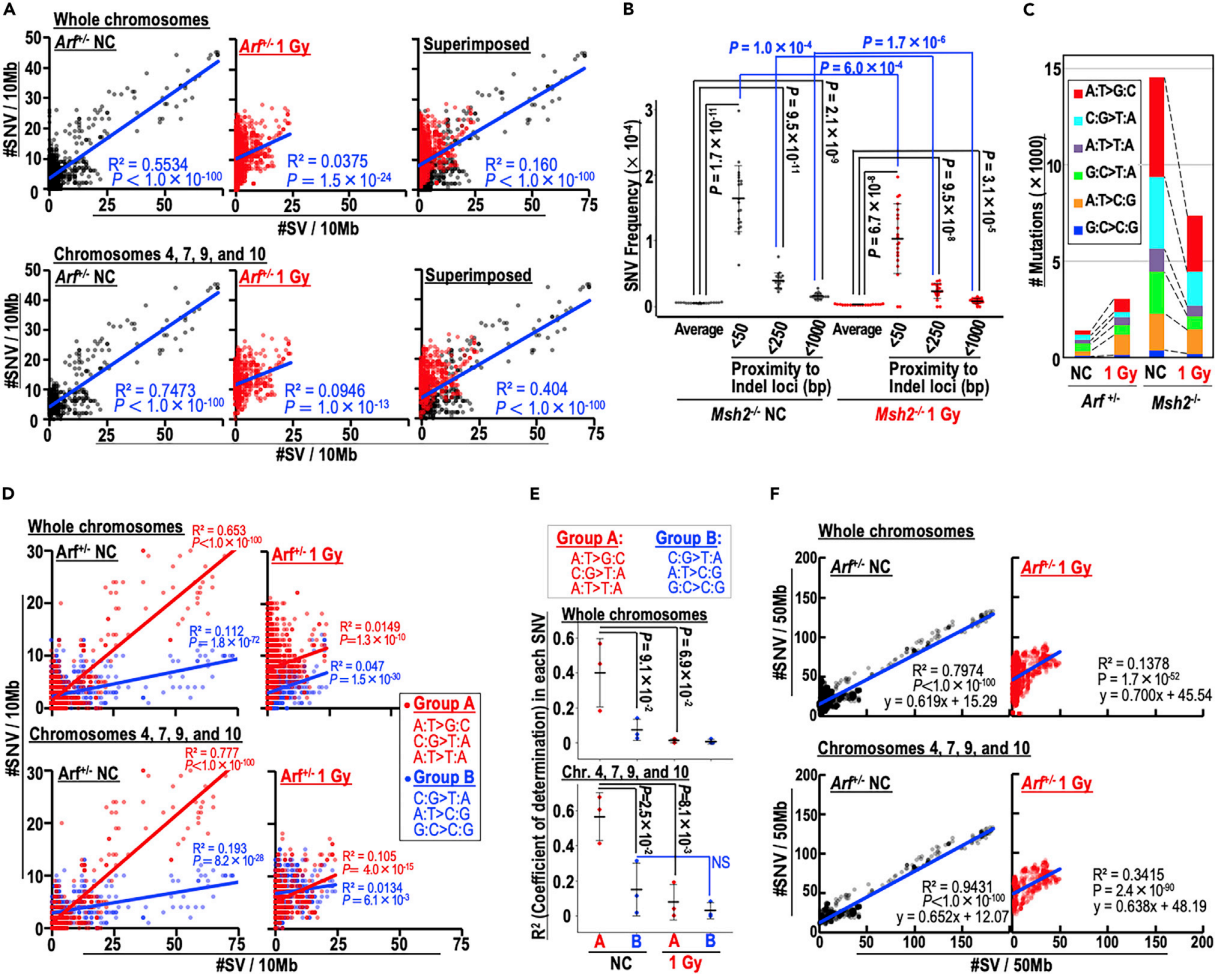
SNV induction is tightly correlated with SVs in both irradiated and non-irradiated backgrounds (A) Correlations between SNV and SV induction were evaluated in 10 Mb intervals for all chromosomes (top) and for chromosomes 4, 7, 9, and 10 (bottom). Student's t test was used for statistical analysis. (B) Correlations between indels and SNVs were evaluated by counting SNV frequencies within 50; 250; and 1,000 bases from indel loci and comparing them to the averages. Two-tailed Welch's t test was used for statistical analysis. (C) Detected mutations were categorized according to the indicated types. (D) Correlations between SNV and SV numbers were determined in 10 Mb intervals for all chromosomes (top) and for chromosomes 4, 7, 9, and 10 (bottom). SNVs and SVs were counted separately in groups A (red) and B (blue). Student's t test was used for statistical analysis. NS, not significant. (E) R^2^ values of linear fitting in each SNV type were separately analyzed, and differences between groups and irradiation backgrounds were determined for all chromosomes (top) and for chromosomes 4, 7, 9, and 10 (bottom). Paired t test was used for statistical analysis within the same group. Two-tailed Welch's t test was used for statistical analysis between groups. NS, not significant. (F) Correlation between SNV and SV inductions was determined in 50 Mb intervals for all chromosomes (top) and for chromosomes 4, 7, 9, and 10 (bottom). Student's t test was used for statistical analysis. See also Figure S5.

Intriguingly, high levels of SNVs were induced at SV hotspots (Figures 4A and 4B: see peaks in Chr. 4). This association was observed over intervals on the order of 10 Mb, unlike kataegis, i.e., a clustered hypermutation (Nik-Zainal et al., 2012) (Figure S5A). The correlation between SV and SNV induction was apparent in the non-irradiated MEFs but dramatically reduced in irradiated MEFs (Figure 5A). Similar results were also observed in *Msh2*^−/−^ MEFs, in which indels (reflecting MSI) were induced as an alternative to CIN through erroneous repair of rs-DSBs (Matsuno et al., 2019). The SNV rate within 50 bases from indel loci was about 30 times higher than average; however, the SNV rate significantly decreased in irradiated MEFs (Figure 5B). These results indicate that SNVs are generally induced in association with genomic destabilization, i.e., SV and indel induction, and that SV-associated SNV levels are reduced in an irradiated background.

We categorized the mutations observed in this study into three signatures. Signature 1 was similar to a spectrum arising under MMR deficiency (Alexandrov et al., 2013), as expected in *Msh2*^−/−^ MEFs (Figures S5C–S5E; see correlation with COSMIC 26). Signature 2 was elevated in the irradiated MEFs. In *Arf*^+/−^ MEFs, the total abundance of SNVs (especially A:T mutations) was increased by irradiation (Figures 5C and S5B), suggesting radiation-associated SNV induction. Analyzing those mutation types, we observed significantly higher SV–SNV correlations in Group A (A:T > C:C, C:G > T:A, and A:T > T:A) than in the other groups and a decrease in such correlations in the irradiated background (Figures 5D and 5E). However, despite the decrease in SV-associated SNVs after irradiation, the correlation was still statistically significant (Figures 5A, 5D, and S5F). To clarify whether those apparent decreases were really due to a decrease in the correlation, or simply reflected the decrease in SV frequency, we further analyzed those associations in a wider range (50 Mb). We observed the tight correlation in SV and SNV induction even in irradiated MEFs (Figure 5F). Together, our results indicate that most SNVs, including those arising due to irradiation, are induced in association with SV/Indel induction. Although SV numbers could be reduced in irradiated backgrounds, most SNVs are induced in association with SV induction.

### cGAS/STING-pathway activation under 10 Gy irradiation

Next, we sought to determine how cells irradiated with higher doses (e.g., 10 Gy) are prevented from immortalization (Figure 1). Because 10 Gy-irradiated cells were continuously arrested from cell-cycle progression and exhibited a flattened and enlarged morphology (Figure S2D), as usually seen in senescent cells (Gorgoulis et al., 2019), we first monitored damage statuses and responses associated with senescence induction, comparing the effects of 1 and 10 Gy irradiation. Unlike at 1 h after irradiation, the number of γH2AX foci did not significantly differ at 48 h after 10 Gy irradiation versus 1 Gy irradiation (Figure S6A). This indicates that damage statuses at later time points are unaffected by the radiation dose, implying that differences in cellular characteristics according to the radiation dose are independent of the resulting damage statuses. In support of this argument, expression of p21, which mediates the p53-dependent senescence-associated phenotype in response to DSBs (Glück et al., 2017), was much weaker 48 h after higher-dose irradiation (4–16 Gy) than after lower dose irradiation (0.25–1 Gy) (Figure S6B). Thus, the effects of the DSB response through p53 and p21 are probably limited to the expression of the 10 Gy-irradiated cellular phenotypes.

**Figure 6:**
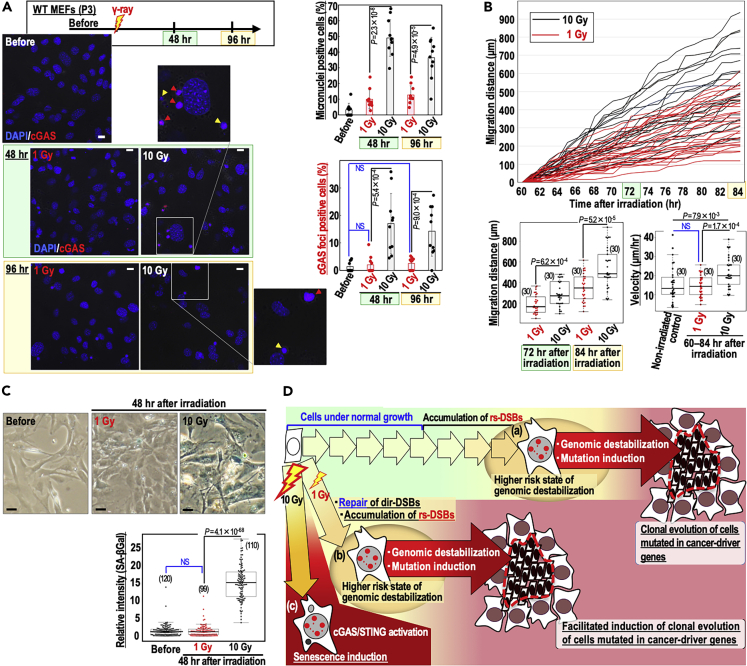
MEFs irradiated with 10 Gy γ-rays undergo senescence in association with the cGAS/STING-pathway activation, unlike MEFs irradiated with 1 Gy (A) After WT MEFs were treated as shown in the workflow, micronuclei and cGAS-foci-positive micronuclei levels were counted (n = 10 independent experiments). Red and yellow arrowheads indicate micronucleus foci with and without co-localized cGAS foci, respectively. Scale bars in images, 10 μm. Quantifications of foci at the indicated time points are shown. Bars show means ± s.d. Two-tailed Welch's t test was used for statistical analysis. NS, not significant. (B) Migration of WT MEFs irradiated with γ-ray (1 or 10 Gy) was analyzed at the indicated time points (top panel); the migration of each cell is indicated by individual lines. Migrated distance observed was plotted for the indicated time points (bottom left panel). Migration velocities of each cell were plotted (bottom right panel). Box plots show the median, third, and first quartiles; whiskers (median ±1.5 times interquartile range); and outliers. Two-tailed Welch's t test was used for statistical analysis. NS, not significant. (C) SA-β-Gal activity of WT MEFs irradiated with γ-rays (1 or 10 Gy). Representative images are shown with quantifications. Scale bars, 10 μm. Box plots show the median, third, and first quartiles; whiskers (median ±1.5 times interquartile range); and outliers. Two-tailed Welch's t test was used for statistical analysis. NS, not significant. (D) Models. After serial cultivation, MEFs enter a state at higher risk of genomic destabilization (a), in which clonal evolution of cells harboring mutations in cancer-driver genes is subsequently induced in association with genomic destabilization. Cells repair dir-DSBs caused by 1 Gy γ-ray and accumulate rs-DSBs during the subsequent S phase, thus entering a cellular state associated with an elevated probability of genomic destabilization (b) and risking clonal evolution induction of cells with mutated cellular defense systems. Unlike cells irradiated with 1 Gy γ-ray (b), cells irradiated with 10 Gy γ-ray enter permanent growth arrest (cellular senescence), which is induced in association with activation of the cGAS/STING pathway (c). See also Figure S6.

Senescence-associated phenotypes could also be induced through activation of the cGAS/STING pathway in response to micronuclei that usually arise with genomic destabilization (Dou et al., 2017; Glück et al., 2017). To explore this possibility, we specifically monitored cytosolic cGAS foci and the associated increases in cellular migration (Bakhoum et al., 2018) and SA-β-Gal activity (Glück et al., 2017). Concomitant with significant increases in the frequency of aberrant micronuclei following 10 Gy irradiation (Figure S6C), the levels of cytosolic cGAS foci were significantly higher in 10 Gy-irradiated MEFs than in 1 Gy-irradiated MEFs (Figure 6A). Consistent with this, 10 Gy-irradiated MEFs exhibited significantly higher migration (Figure 6B and Videos S4, S5, and S6) and SA-β-Gal activity (Figure 6C). Activation of the cGAS/STING pathway may trigger bystander effects via secreted factors (Liao et al., 2014). Therefore, we tested the effects of conditioned medium on cellular migration as shown in the workflow in Figure S6D. Cellular migration was significantly increased by conditioned medium of 10 Gy-irradiated cells but not of 1 Gy-irradiated cells. These results imply the involvement of bystander effects (Figure S6D). Together, MEFs irradiated with 10 Gy γ-rays exhibit senescence-associated phenotypes mainly through cGAS/STING-pathway activation, ultimately suppressing clonal evolution.

Video S4. WT MEFs (P3) irradiated with 1 Gy γ-rays, related to Figure 6

Video S5. WT MEFs (P3) irradiated with 10 Gy γ-rays, related to Figure 6

Video S6. WT MEFs (P3) without irradiation, related to Figure 6

## Discussion

The findings of this study revealed that among the many types of damage, stress, and adducts caused by radiation, rs-DSBs that trigger genomic destabilization are largely responsible for cancer-driver mutations and resulting clonal evolution. Even without irradiation, rs-DSBs often accumulate after serial cell proliferation, risking genomic destabilization and ultimately clonal evolution of cells harboring mutations in cellular defense systems (Matsuno et al., 2019) (Figure 6D; see cellular state a). A primary effect of radiation is induction of a cellular state at higher risk of genomic destabilization due to the accumulation of rs-DSBs after repair of dir-DSBs (Figure 6D; see cellular state b), and the subsequent effects are identical to those in cellular state A. Cellular state B is induced by a wide range of radiation doses (0.25–2 Gy) and dose rates (1.39–909 mGy/min). By contrast, cells exposed to higher doses (e.g., 10 Gy) exhibit continuous cell-cycle arrest and senescence-associated phenotypes mainly through the cGAS/STING-pathway activation, which ultimately blocks clonal evolution (Figure 6D; see cellular state c).

Our results revealed induction of SV/indel-associated SNVs even in an irradiated background in which radiation-associated SNVs (mutations in A:T base pairs) are also increased. Therefore, irradiated cells that are at higher risk of genomic destabilization are simultaneously at higher risk of SNV induction, risking clonal evolution of cells with abrogated defense systems. Mutations in cancer-suppressor genes could be caused by SV-associated SNV induction, as well as SV-mediated genetic alterations, such as gene-locus deletion and LOH induction.

Cancer is generally caused by cancer-driver mutations. Although it was once believed that most errors are randomly induced during canonical replication (Tomasetti et al., 2017), this might not be the case. In fact, a recent study revealed another pathway: genomic destabilization-associated mutagenesis (Matsuno et al., 2019). Importantly, although errors during canonical replication are limited even in MMR-deficient cells, which cannot repair replication errors, massive mutations (including cancer-driver mutations) are induced in association with genomic destabilization because low-fidelity TLS polymerases are mainly operating in that state (Matsuno et al., 2019). Supporting this argument, we observed a tight correlation in SV and SNV induction (Figures 4A, 4B, and 5). In addition, our findings revealed that radiation exposure increases the risk of genomic destabilization with associated mutagenesis.

The effects of radiation have been studied for many years, and an important longstanding question is how radiation increases cancer risk. Based on our results, we propose that risk is primarily associated with induction of a cellular state with a greater likelihood of genomic destabilization owing to the accumulation of rs-DSBs but not to lesions directly caused by radiation. This conclusion is supported by multiple results. (1) The abundances of SV and indels are not increased by irradiation, and are even decreased under an irradiated background (Figures 1, 2, 3, 4D, and 4E). (2) The levels of rs-DSBs that trigger CIN induction are not increased by irradiation dose or dose rate (Figures 1, 2, and 3). (3) Although genomic destabilization could be massively induced by higher-dose irradiation (e.g., 10 Gy) (Figure S6C), such effects are associated with the suppression of clonal evolution by induction of cellular senescence through the cGAS/STING-pathway activation (Figure 6).

How do rs-DSBs arise upon exposure to γ-ray irradiation? Ionizing radiation generally causes clustered DNA lesions of oxidatively generated base damage, abasic damage, SSBs, and dir-DSBs (Georgakilas et al., 2013; Nickoloff et al., 2020; Sage and Shikazono, 2017). Although those lesions other than dir-DSBs can cause rs-DSBs, this is probably not the major pathway because these lesions are usually repairable within a few hours, whereas most rs-DSBs are induced much later, i.e., 24–48 h after irradiation. Moreover, SVs are induced in the same hotspots in irradiated and non-irradiated cells (Figures 4A and 4C), implying that rs-DSB formation is independent of lesions directly caused by irradiation. Radiation damage is widely generated by oxidation; therefore, resulting rs-DSB accumulation might be caused via effects of oxidation other than those on DNA, such as generation of H_2_O_2_ and oxidated nucleotides and proteins. Given that endogenous DNA lesions inevitably arise at a rate of as high as 20,000/cell/day (Barnes and Lindahl, 2004), rs-DSBs might accumulate when the level of lesions exceeds the repairable limit owing to additional irradiation-associated lesions.

Unlike effectively repairable dir-DSBs, continuously arising rs-DSBs were induced over wide ranges of radiation doses (0.25–2 Gy) and dose rates (1.39–909 mGy/min). These observations are consistent with previous reports showing two types of radiation-induced DSBs, i.e., repairable and unrepairable DSBs, in which the latter are observed even at very low doses (e.g., 10 mGy) (Grudzenski et al., 2010; Sedelnikova et al., 2004). As this study revealed, such persistent rs-DSBs risk further genomic destabilization and associated mutagenesis. However, the resultant acceleration in clonal evolution is only apparent when the MEFs are irradiated in the growing state but not the cell-cycle-arrested state (Figure S6E), implying that cycling cells, but not dormant cells, are sensitive to this risk. This is analogous to the situation for radiation-associated cancers, which are significant risk factors in infants but not in adults (Gilbert, 2009), probably due to the comparatively large population of actively cycling stem cells early in life.

### Limitations of the study

We revealed that rs-DSBs caused by γ-ray irradiation risk genomic destabilization and hence lead to clonal evolution in MEF model. Although our results give the mechanistic insight of radiation risk for cancer development, further experiments are necessary to understand how cancer is induced by γ-ray irradiation *in vivo*.

### Resource availability

#### Lead contact

Further information and requests for resource should be directed to and will be fulfilled by the lead contact Ken-ichi Yoshioka (kyoshiok@ncc.go.jp).

#### Materials availability

This study did not generate new unique reagents.

#### Data and code availability

The sequencing data obtained by whole genome analyses were deposited in the DDBJ database (under the accession number DRA011651).

## Methods

All methods can be found in the accompanying transparent methods supplemental file.

## Supplemental information

Document S1. Transparent methods and Figures S1–S6

## References

[bib1] Alexandrov L.B., Nik-Zainal S., Wedge D.C., Aparicio S.A.J.R., Behjati S., Biankin A.V., Bignell G.R., Bolli N., Borg A., Børresen-Dale A.L. (2013). Signatures of mutational processes in human cancer. Nature.

[bib2] Atsumi Y., Fujimori H., Fukuda H., Inase A., Shinohe K., Yoshioka Y., Shikanai M., Ichijima Y., Unno J., Mizutani S. (2011). Onset of Quiescence following p53 mediated down-regulation of H2AX in normal cells. PLoS One.

[bib3] Atsumi Y., Minakawa Y., Ono M., Dobashi S., Shinohe K., Shinohara A., Takeda S., Takagi M., Takamatsu N., Nakagama H. (2015). ATM and SIRT6/SNF2H mediate transient H2AX stabilization when DSBs form by blocking HUWE1 to allow efficient γH2AX foci formation. Cell Rep..

[bib4] Bakhoum S.F., Ngo B., Laughney A.M., Cavallo J.A., Murphy C.J., Ly P., Shah P., Sriram R.K., Watkins T.B.K., Taunk N.K. (2018). Chromosomal instability drives metastasis through a cytosolic DNA response. Nature.

[bib5] Barnes D.E., Lindahl T. (2004). Repair and genetic consequences of endogenous DNA base damage in mammalian cells. Annu. Rev. Genet..

[bib6] Borrego-Soto G., Ortiz-López R., Rojas-Martínez A., Borrego-Soto G., Ortiz-López R., Rojas-Martínez A. (2015). Ionizing radiation-induced DNA injury and damage detection in patients with breast cancer. Genet. Mol. Biol..

[bib7] Burhans W.C., Weinberger M. (2007). DNA replication stress, genome instability and aging. Nucleic Acids Res..

[bib8] Burtt J.J., Thompson P.A., Lafrenie R.M. (2016). Non-targeted effects and radiation-induced carcinogenesis: a review. J. Radiol. Prot..

[bib9] Dou Z., Ghosh K., Vizioli M.G., Zhu J., Sen P., Wangensteen K.J., Simithy J., Lan Y., Lin Y., Zhou Z. (2017). Cytoplasmic chromatin triggers inflammation in senescence and cancer. Nature.

[bib10] Georgakilas A.G., O’Neill P., Stewart R.D. (2013). Induction and repair of clustered DNA lesions: what do we know so far?. Radiat. Res..

[bib11] Gilbert E.S. (2009). Ionising radiation and cancer risks: what have we learned from epidemiology?. Int. J. Radiat. Biol..

[bib12] Glück S., Guey B., Gulen M.F., Wolter K., Kang T.-W., Schmacke N.A., Bridgeman A., Rehwinkel J., Zender L., Ablasser A. (2017). Innate immune sensing of cytosolic chromatin fragments through cGAS promotes senescence. Nat. Cell Biol..

[bib13] Gorgoulis V., Adams P.D., Alimonti A., Bennett D.C., Bischof O., Bishop C., Campisi J., Collado M., Evangelou K., Ferbeyre G. (2019). Cellular senescence: defining a path forward. Cell.

[bib14] Grudzenski S., Raths A., Conrad S., Rübe C.E., Löbrich M., Grudzenski S., Raths A., Conrad S., Rübe CE L.M. (2010). Inducible response required for repair of low-dose radiation damage in human fibroblasts. Proc. Natl. Acad. Sci. U S A.

[bib15] Howe G., Bouville A., Shibata Y., Reiners C., Repacholi M., Gudzenko N., Thomas G., Davis S., Kesminiene A., Kopecky K.J. (2006). Cancer consequences of the Chernobyl accident: 20 years on. J. Radiol. Prot..

[bib16] Ichijima Y., Yoshioka K., Yoshioka Y., Shinohe K., Fujimori H., Unno J., Takagi M., Goto H., Inagaki M., Mizutani S. (2010). DNA lesions induced by replication stress trigger mitotic aberration and tetraploidy development. PLoS One.

[bib17] Liao E.C., Hsu Y.T., Chuah Q.Y., Lee Y.J., Hu J.Y., Huang T.C., Yang P.M., Chiu S.J. (2014). Radiation induces senescence and a bystander effect through metabolic alterations. Cell Death Dis..

[bib18] Lomax M.E., Folkes L.K., O’Neill P. (2013). Biological consequences of radiation-induced DNA damage: relevance to radiotherapy. Clin. Oncol..

[bib19] Matheu A., Maraver A., Klatt P., Flores I., Garcia-Cao I., Borras C., Flores J.M., Viña J., Blasco M.A., Serrano M. (2007). Delayed ageing through damage protection by the Arf/p53 pathway. Nature.

[bib20] Matsuno Y., Atsumi Y., Shimizu A., Katayama K., Fujimori H., Hyodo M., Minakawa Y., Nakatsu Y., Kaneko S., Hamamoto R. (2019). Replication stress triggers microsatellite destabilization and hypermutation leading to clonal expansion in vitro. Nat. Commun..

[bib21] Minakawa Y., Atsumi Y., Shinohara A., Murakami Y., Yoshioka K. (2016). Gamma-irradiated quiescent cells repair directly induced double-strand breaks but accumulate persistent double-strand breaks during subsequent DNA replication. Genes Cells.

[bib22] Mirzayans R., Andrais B., Scott A., Murray D. (2012). New insights into p53 signaling and cancer cell response to DNA damage: implications for cancer therapy. J. Biomed. Biotechnol..

[bib23] Morgan W.F., Sowa M.B. (2015). Non-targeted effects induced by ionizing radiation: mechanisms and potential impact on radiation induced health effects. Cancer Lett..

[bib24] Nickoloff J.A., Sharma N., Taylor L. (2020). Clustered DNA double-strand breaks: biological effects and relevance to cancer radiotherapy. Genes (Basel).

[bib25] Nik-Zainal S., Alexandrov L.B., Wedge D.C., Van Loo P., Greenman C.D., Raine K., Jones D., Hinton J., Marshall J., Stebbings L.A. (2012). Mutational processes molding the genomes of 21 breast cancers. Cell.

[bib26] Osawa T., Atsumi Y., Sugihara E., Saya H., Kanno M., Tashiro F., Masutani M., Yoshioka K. (2013). Arf and p53 act as guardians of a quiescent cellular state by protecting against immortalization of cells with stable genomes. Biochem. Biophys. Res. Commun..

[bib27] Sage E., Shikazono N. (2017). Radiation-induced clustered DNA lesions: repair and mutagenesis. Free Radic. Biol. Med..

[bib28] Sedelnikova O.A., Horikawa I., Zimonjic D.B., Popescu N.C., Bonner W.M., Barrett J.C. (2004). Senescing human cells and ageing mice accumulate DNA lesions with unrepairable double-strand breaks. Nat. Cell Biol..

[bib29] Shiloh Y., Ziv Y. (2013). The ATM protein kinase: regulating the cellular response to genotoxic stress, and more. Nat. Rev. Mol. Cell Biol..

[bib30] Suzuki K., Ojima M., Kodama S., Watanabe M. (2003). Radiation-induced DNA damage and delayed induced genomic instability. Oncogene.

[bib31] Toledo L.I., Altmeyer M., Rask M.-B., Lukas C., Larsen D.H., Povlsen L.K., Bekker-Jensen S., Mailand N., Bartek J., Lukas J. (2013). ATR prohibits replication catastrophe by preventing global exhaustion of RPA. Cell.

[bib32] Tomasetti C., Li L., Vogelstein B. (2017). Stem cell divisions, somatic mutations, cancer etiology, and cancer prevention. Science.

[bib33] Yoshioka K., Atsumi Y., Fukuda H., Masutani M., Teraoka H. (2012). The quiescent cellular state is Arf/p53-dependent and associated with H2AX downregulation and genome stability. Int. J. Mol. Sci..

[bib34] Zeman M.K., Cimprich K.A. (2014). Causes and consequences of replication stress. Nat. Cell Biol..

