# Supplementary material for: Replication-stress-associated DSBs induced by ionizing radiation risk genomic destabilization and associated clonal evolution
Source: iScience. 2021 Mar 15;24(4):102313. doi: 10.1016/j.isci.2021.102313 (PMC8042347; doi:10.1016/j.isci.2021.102313)
Supplement: Document S1. Transparent methods and Figures S1–S6 [file mmc1.pdf]

iScience, Volume *24*

Supplemental information

Replication-stress-associated DSBs induced by ionizing radiation risk genomic destabilization and associated clonal evolution

Yusuke Matsuno, Mai Hyodo, Mafuka Suzuki, Yosuke Tanaka, Yasunori Horikoshi, Yasufumi Murakami, Hidetaka Torigoe, Hiroyuki Mano, Satoshi Tashiro, and Ken-ichi Yoshioka

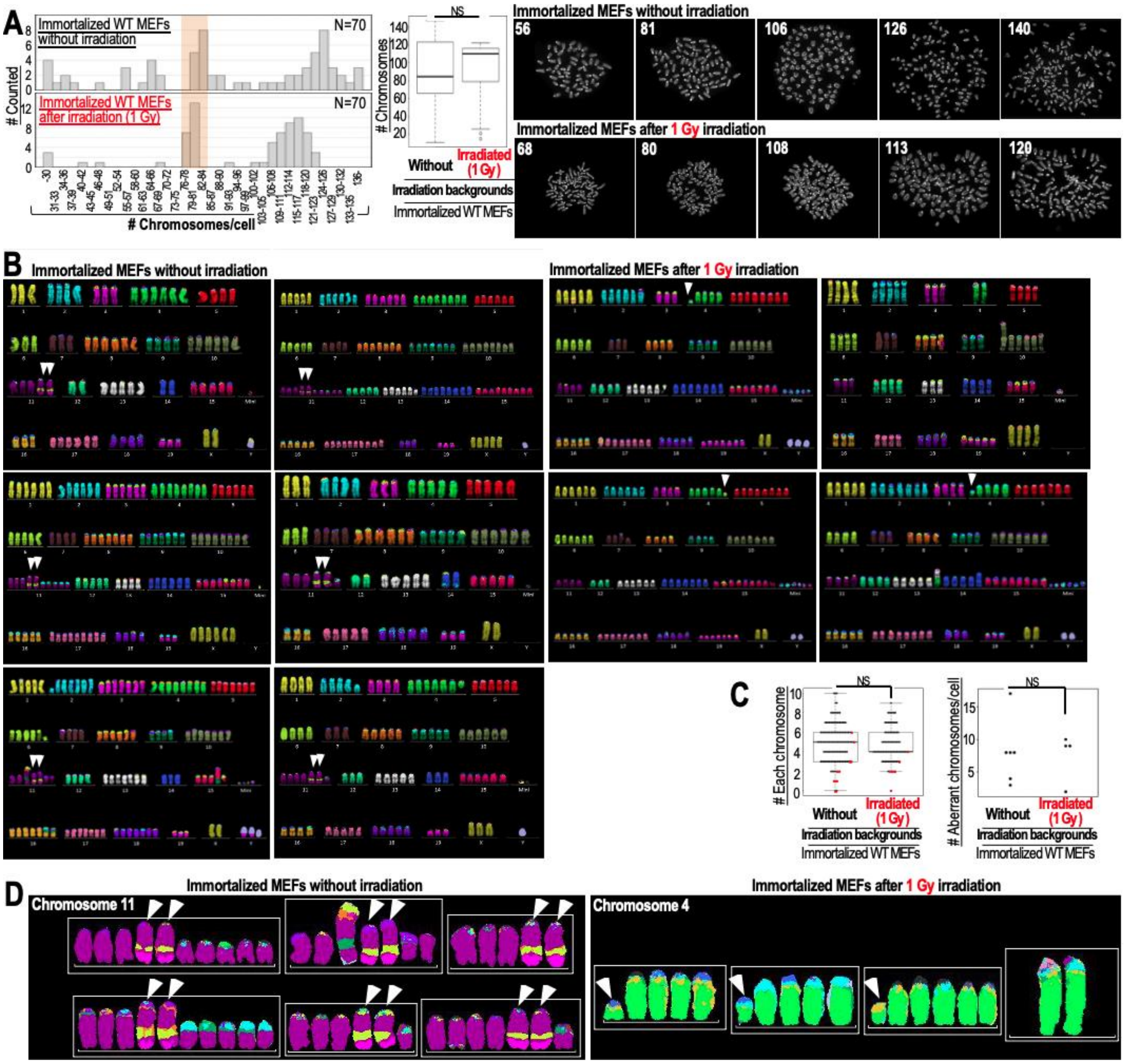

Figure S1. Chromosomal abnormalities are induced in immortalized WT MEFs in both 1 Gy-irradiated and non-irradiated background, related to Figure 1.

(A, B) Representative images of M-phase chromosomes after H33258 staining (**A**) and multicolor FISH (**B**); karyotyping images are also shown (**B**). Box plots show median, third, and first quartiles; whiskers (median ± 1.5 times interquartile range); and outliers. Two-tailed Welch's *t*-test was used for statistical analysis. NS, not significant.

(C) Left panel: copy number of each chromosome per cell. X- and Y-chromosomes are indicated by red dots. Right panel: number of abnormal chromosomes other than centromeres (n = 6 immortalized MEFs without irradiation and n = 44 immortalized

MEFs after 1 Gy irradiation). Box plots show median, third, and first quartiles; whiskers (median ± 1.5 times interquartile range); and outliers. Two-tailed Welch's *t*-test was used for statistical analysis. NS, not significant.

(D) Chromosomal translocations or deletions uniformly observed in multiple cells are indicated by white arrowheads.

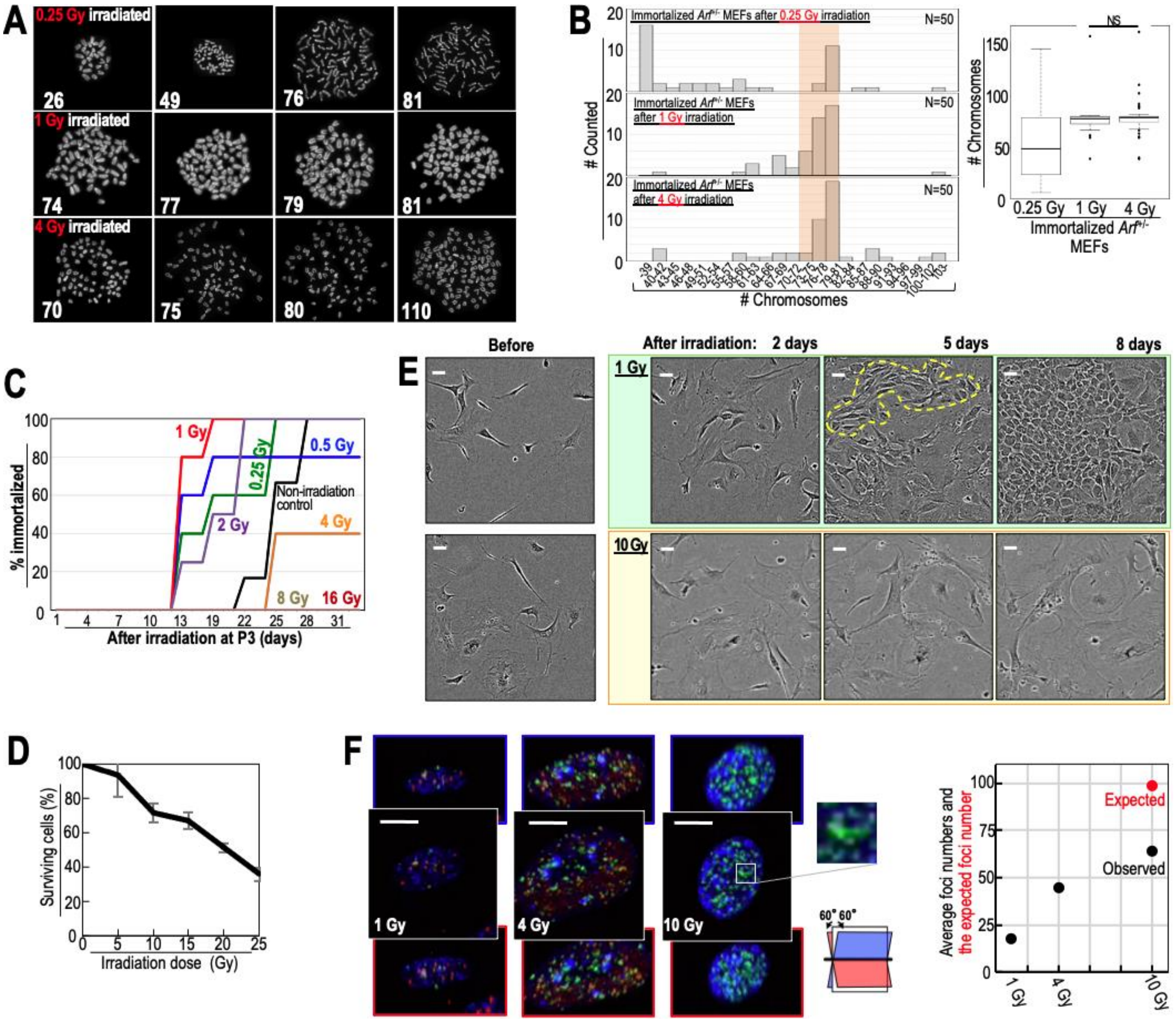

Figure S2. Genomic destabilization-associated immortalization is induced after irradiation with a wide range of doses, related to Figure 2.

(A) Representative M-phase chromosome images of immortalized *Arf*$^{+/-}$ MEFs after visualization by H33258 staining.

(B) CIN induction status was determined by chromosome number analysis. Box plots show the median, third, and first quartiles; whiskers (median ± 1.5 times interquartile range); and outliers. Two-tailed Welch's *t*-test was used for statistical analysis. NS indicates not significant.

(C) Multiple subcultures of 1×10^4 *Arf*$^{+/-}$ MEFs (P3) were seeded for analysis of immortalization speed.

(D) Surviving *Arf*$^{+/-}$ MEFs were counted 1 week after γ-ray irradiation and the percentage of these cells compared with untreated cells was plotted. The graph shows

mean survival rates ± s.d. (n = 3 independent experiments with MEFs prepared from independent fetuses).

(E) Time-lapse image analyses were performed on MEFs treated as in **Figure 2A**. Representative images are shown. These results are also shown in the accompanying movies [Supplementary movies S1 (non-irradiated control), S2 (MEFs irradiated with 1 Gy γ-rays), and S3 (MEFs irradiated with 10 Gy γ-rays)]. Scale bars in images, 10 μm.

(F) *Arf*$^{+/-}$ MEFs were irradiated with γ-rays, and γH2AX/53BP1 foci were analyzed 1 hr later. Representative images are provided together with 60° tilted images. Scale bars in images, 10 μm. After evaluation of the numbers of apparent γH2AX foci, the average numbers of these foci were plotted (right panel). The observed number of γH2AX foci was lower than expected in 10 Gy-irradiated MEFs, largely because some of these foci were not clearly separated.

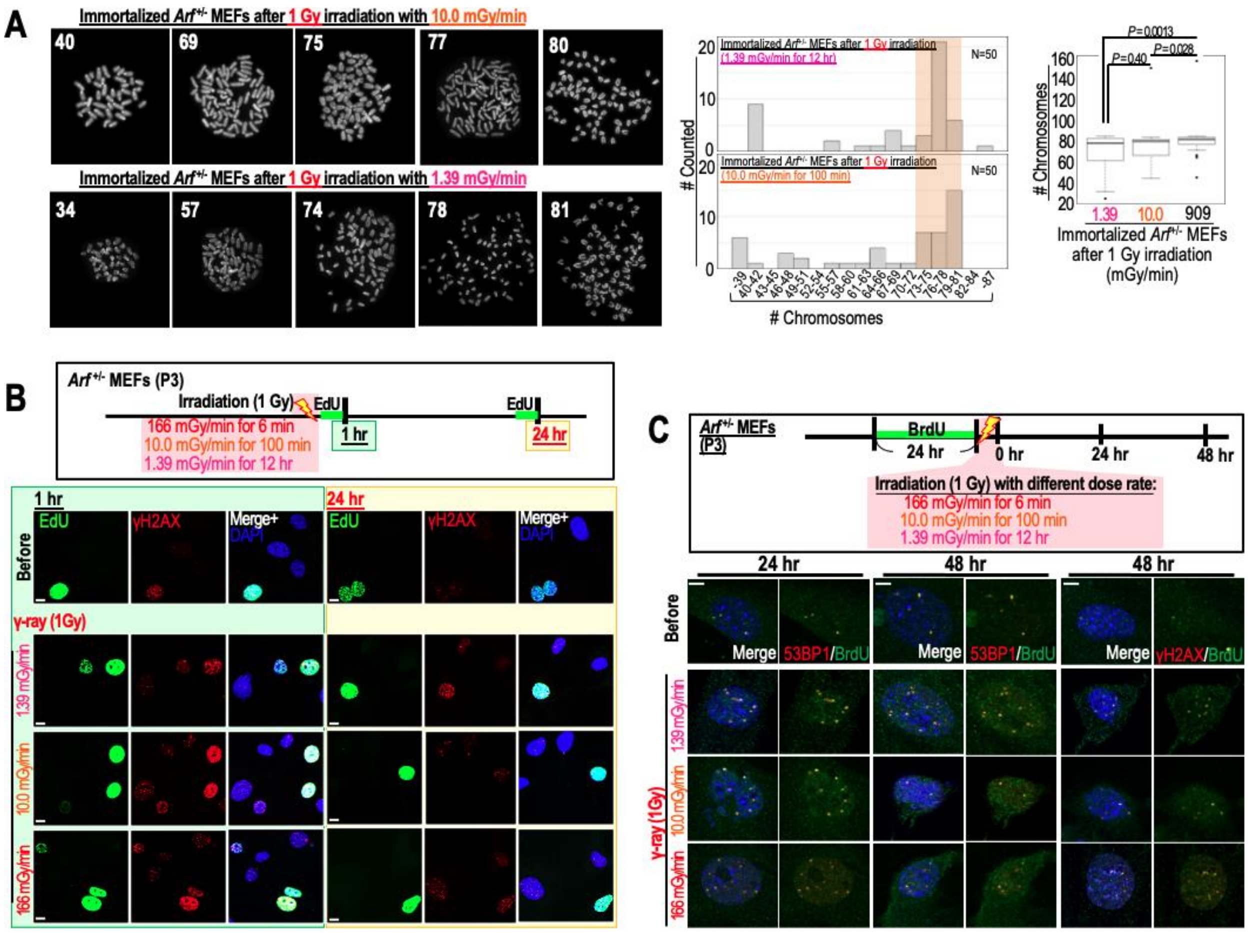

Figure S3. Induction of abnormal chromosome number in association with rs-DSB accumulation due to irradiation at a wide range of dose rates, related to Figure 3.

(A) Representative M-phase chromosome images of immortalized *Arf*$^{+/-}$ MEFs after H33258 staining. Box plots show the median, third, and first quartiles; whiskers (median ± 1.5 times interquartile range); and outliers. Two-tailed Welch's *t*-test was used for statistical analysis.

(B) *Arf*$^{+/-}$ MEFs were treated as shown in the workflow. EdU-positive MEFs were co-immunostained for γH2AX. Representative images are provided. Scale bars in images, 10 μm.

(C) *Arf*$^{+/-}$ MEFs were treated as shown in the workflow. Co-localized foci of γH2AX/BrdU and 53BP1/BrdU were detected by immunofluorescence under native conditions. Scale bars, 2 μm.

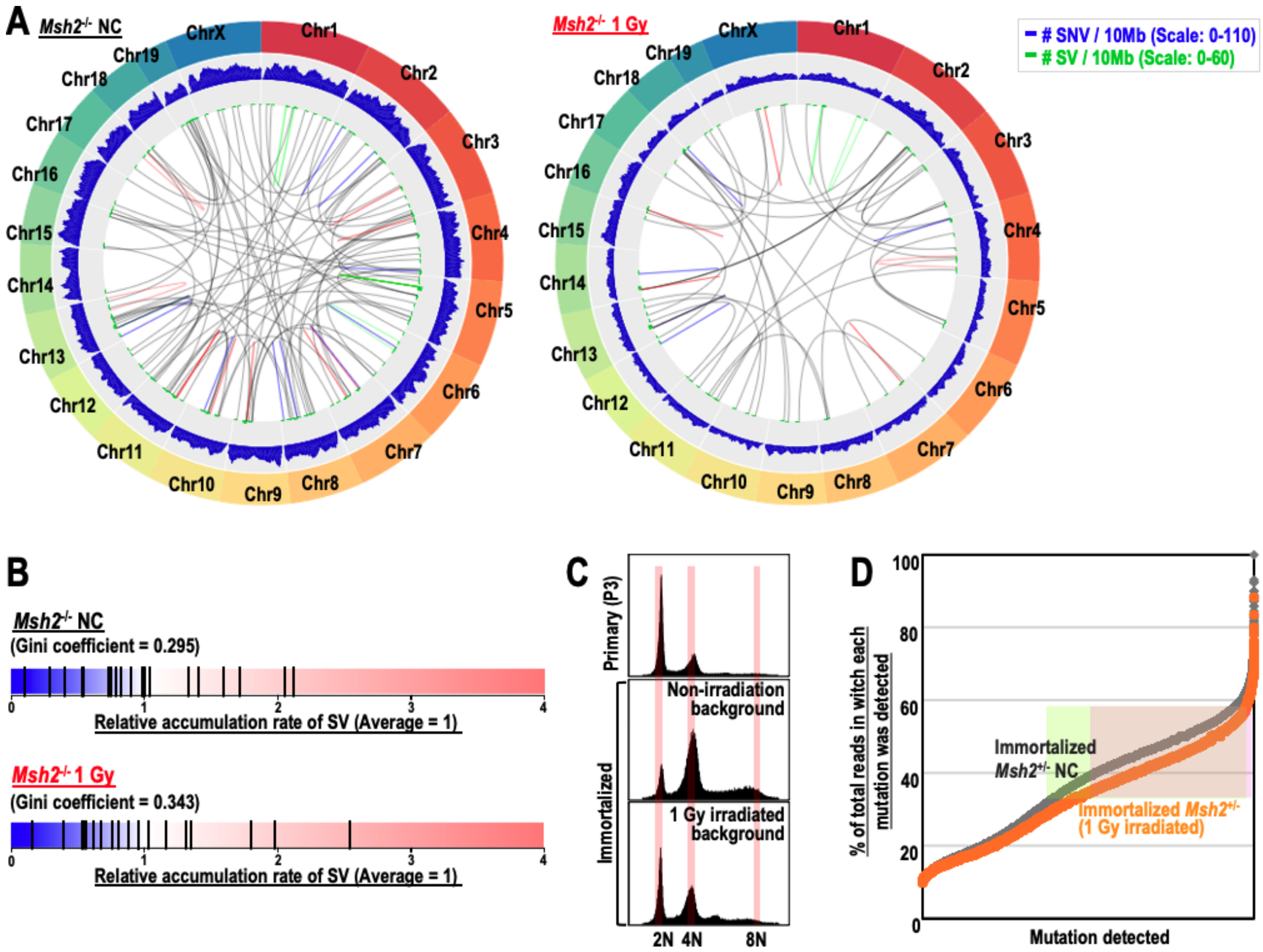

Figure S4. SV in immortalized *Msh2*$^{-/-}$ MEFs is induced under both 1 Gy-irradiated and non-irradiated backgrounds, related to Figure 4.

(A) Genome-wide Circos plots of SVs and SNVs are shown. Chromosome ideograms are shown around the outer ring. The two inner circular tracks show numbers of SVs (green) and SNVs (blue) with the corresponding moving averages. Inside lines indicate duplications (blue lines), inversions (green lines), deletions (red lines), and translocations (black lines).

(B) Relative SV accumulation rates in each chromosome.

(C) Ploidy statuses of immortalized *Arf*$^{+/-}$ MEFs were determined by flow cytometry.

(D) Mutations detected in immortalized *Msh2*$^{-/-}$ MEFs. The percentage of reads detected as mutations is shown.

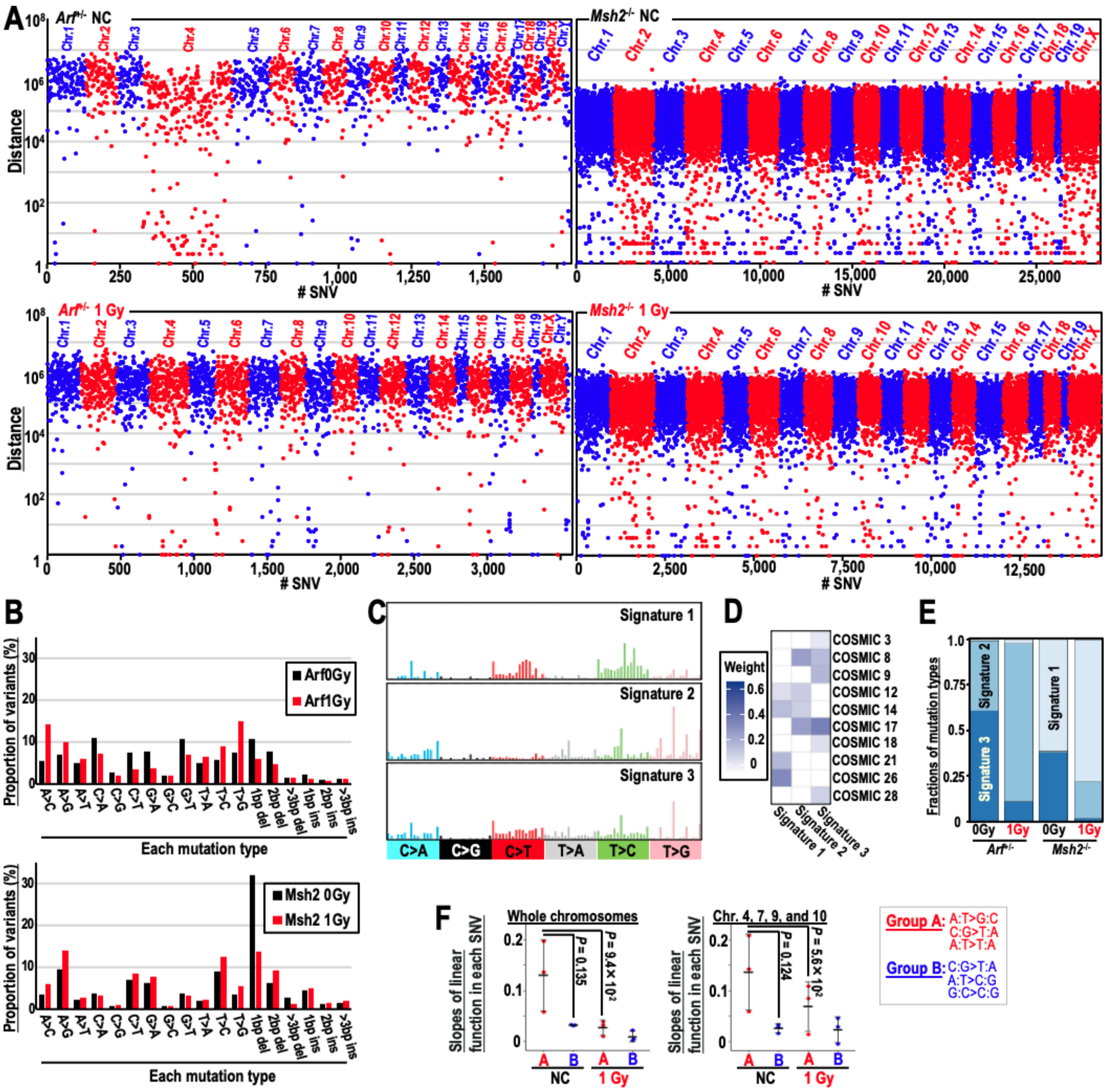

Figure S5. Mutations induced in immortalized *Arf*$^{+/-}$ and *Msh2*$^{-/-}$ MEFs, related to Figure 5.

(A) Mutational profiles are shown as rainfall plots. The x-axis shows mutations, ordered by mutation number; the y-axis represents inter-mutation distance in log scale.

(B) Mutations detected by whole genome sequence were categorized according to the indicated types.

(C)–(E) Three mutational signatures were identified in MEF models **(C)**. Each was compared using COSMIC V2 **(D)**. Fractions of each mutational signature type **(E)**.

(F) Slopes of linear fitting in each SNV type were analyzed separately, and differences in groups and irradiation background were determined. Two-tailed Welch's *t*-test was used for statistical analysis.

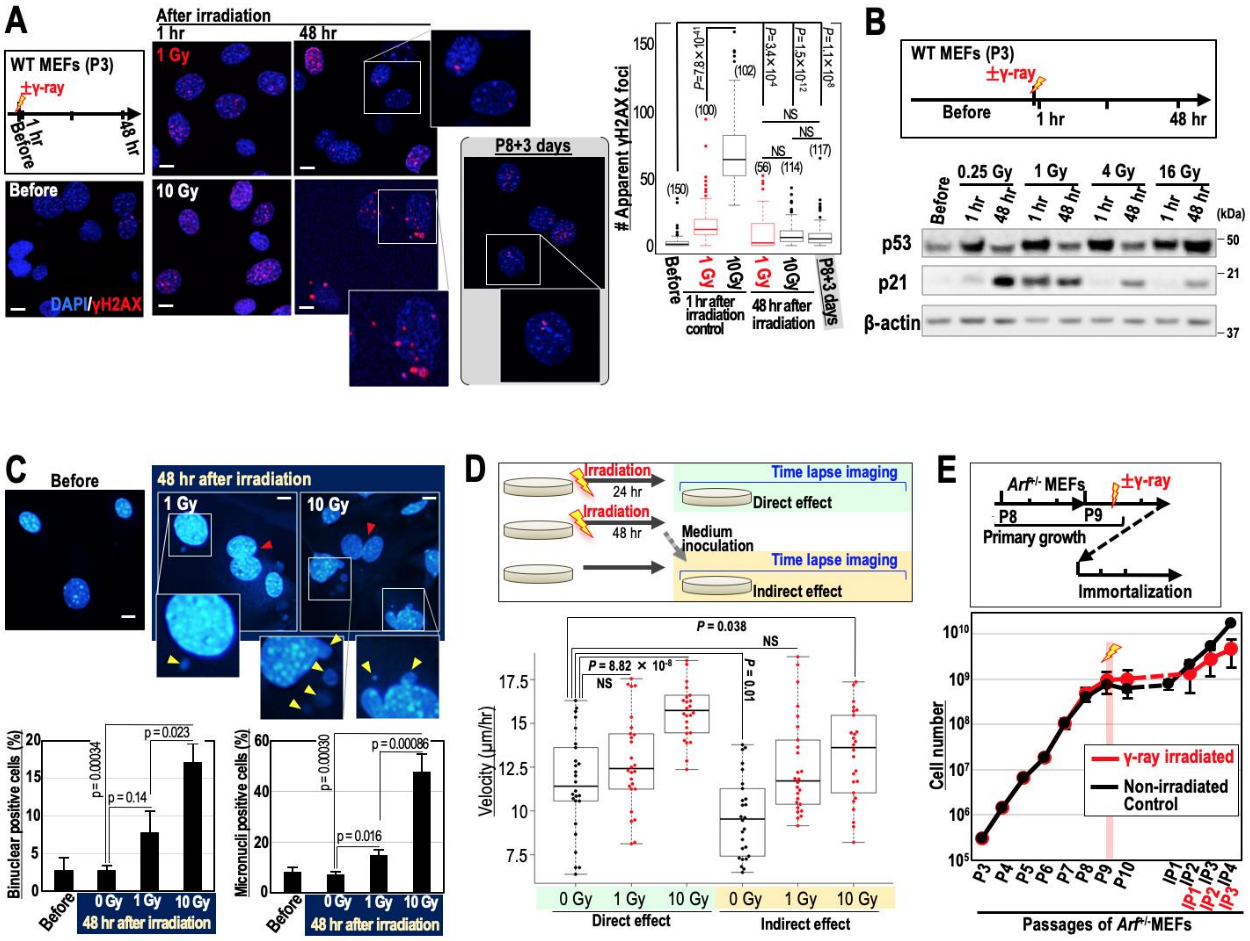

Figure S6. Cells irradiated at higher doses do not accumulate p21 but have aberrant nuclei, related to Figure 6.

(A) WT MEFs were irradiated with γ-ray (1 or 10 Gy) as shown in the workflow, and the levels of γH2AX foci were monitored. MEFs at P8 + 3 days are shown as controls. Scale bars, 10 μm. Box plots show median, third, and first quartiles; whiskers (median ± 1.5 times interquartile range); and outliers. Two-tailed Welch's *t*-test was used for statistical analysis. NS, significant.

(B) WT MEFs were treated as shown in the workflow. Accumulation status of p53 and p21 was assessed by western blotting.

(C) Bi-nuclear and micronuclei status of WT MEFs irradiated with γ-ray (0, 1, or 10 Gy). Scale bars, 10 μm. Bars show means ± s.d. Two-tailed Welch's *t*-test was used for statistical analysis. NS, not significant.

(D) Migration of WT MEFs inoculated with the conditioned media of MEFs irradiated with γ-rays (0, 1, or 10 Gy) was analyzed as shown in the workflow, in comparison with

directly irradiated MEFs. Migration velocities of each cell were plotted. Box plots show the median, third, and first quartiles; whiskers (median ± 1.5 times interquartile range); and outliers. Two-tailed Welch's *t*-test was used for statistical analysis. NS, not significant.

(E) *Arf*$^{+/-}$ MEFs at P9 were irradiated with γ-rays and cultivated under the Std-3T3 protocol to monitor the immortalization process, in comparison with the non-irradiated control. The graph shows mean cell numbers ± s.d. (n = 3 independent experiments with MEFs prepared from independent fetuses).

Transparent Methods

Cell culture. WT and *Arf*$^{+/-}$ MEFs were prepared from embryos of WT or *Arf*$^{+/-}$ mice and cultured under a 3T3 passage protocol. To obtain immortalized MEFs, MEFs that reached the growth-arrested state were maintained in culture, with a medium change every 3 days, until they exhibited immortal growth (IP1). All MEFs were cultured in Dulbecco's modified Eagle's medium supplemented with 10% (v/v) fetal calf serum (FCS).

Flow cytometry, Q-band analysis, and karyotyping. Ploidy status was determined by flow cytometry. For chromosomal abnormality studies (Matsuno et al., 2019), cells cultivated in the presence of 12.5–25 ng/mL colcemid for 1.5–5 hr were trypsinized, collected by centrifugation (1,500 rpm for 5 min), suspended in 75 mM KCl (5 mL), and incubated for 20 min. Carnoy's solution (acetic acid:methanol = 1:3) (6 mL) was added to the cell suspension before centrifugation (1,500 rpm for 5 min). The pellet was washed twice by suspending cells in Carnoy's solution (5 mL), followed by centrifugation (1,500 rpm for 5 min). The resultant chromosome samples were again suspended in Carnoy's solution (0.1–1 mL) and spread onto glass slides. The slides were incubated at 80°C for 48 hr, soaked in 0.025% trypsin prepared in phosphate-buffered saline (PBS) (37°C) for a few seconds, and then washed once in 10% FCS in PBS and once in 5% FCS in PBS. For chromosome counting, the slides were stained by soaking for 30 min in 10 ng/mL bisBenzimide H33258 in McIlvaine buffer (pH 4.4), and subsequently washed with water. After another round of soaking in McIlvaine buffer and washing with water, the slides were dried, exposed to fluorescent light for more than 48 hr, and then embedded in 50% glycerol–McIlvaine buffer. Chromosomes

were monitored by microscopy (Zeiss Axio Imager Z2) and analyzed using the Metafer4 and Ikaros software (MetaSystems). mFISH was performed using a mouse mFISH probe (MetaSystems). Metaphase images were captured using an AxioImagerZ2 fluorescence microscope and the ISIS mFISH software (MetaSystems). For counting of chromosomal translocations, because the centromeric region could be mis-hybridized, translocations other than centromeric regions (Figure 1D) were analyzed separately from those with centromeric translocations, as revealed by the hybridization patterns (Figure S1B).

Cell biological experiments. DNA damage in the experiments shown in Figures 1, 2, and 4 was induced by ^{137}Cs irradiation in a Gammacell 40 Exactor (Best Theratronics) at a dose rate of 909 mGy/min. For dose-rate experiments, cells were exposed to 1 Gy/6 min (166 mGy/min) by ^{137}Cs irradiation in a Gammacell 40 Exactor (Best Theratronics) using a collimator. Dose rates of 1 Gy/100 min (10 mGy/min) and 1 Gy/12 hr (1.39 mGy/min) were administered using a ^{137}Cs-gamma irradiator (Chugai Technos). After proteins were resolved by electrophoresis and transferred to PVDF membranes, western blotting was performed using the antibodies indicated below. For analysis of *Cdkn2a* status in immortalized MEFs, the locus was assayed by electrophoresis after PCR amplification of the fragment. PCR fragments of the *Tp53* and *Gapdh* genes were used as controls. PCR primers for *Tp53* were 5'-ATTAGTTCCCCACCTTGACAC-3' and 5'-TGGCAGAATAGCTTATTGAGGG-3'. PCR for *Cdkn2a* was performed first with 5'-GGACCCGAAAGTTAACCGGAG-3' and 5'-TTGAGGAGGACCGTGAAGCCG-3', and subsequently nested with 5'-AGTACAGCAGCGGGAGCATGG-3' and 5'-GGACCCGAAAGTTAACCGGAG-3'. PCR for *Gapdh* was performed first with 5'-

AACTTTGGCATTGTGGAAGG-3’ and 5’-CTGCTTCACCACCTTCTTGA-3’, and subsequently nested with 5’-AAGGTCATCCCAGAGCTGAA-3’ and 5’-CACATTGGGGGTAGGAACA-3’.

Cell imaging and analyses. Cells were prepared by 4% paraformaldehyde fixation, permeabilization with 0.1% Triton X-100/PBS, and blocking (2% goat serum in PBS containing 0.3% Triton X-100), and then subjected to immunofluorescence using the primary and secondary antibodies indicated below. Immunofluorescence was performed on a confocal laser microscope (Matsuno et al., 2019) (Olympus FV10i and Leica SP8). γH2AX and 53BP1 foci in each nucleus were automatically counted using the “Find Maxima” function of ImageJ after manual identification of nuclei, which were visualized by DAPI staining. Foci counting was performed under the same conditions in each experiment. Although the number of countable foci 1 hr after 10 Gy irradiation was lower than expected because these foci were not always clearly separated (Figure S2F), foci in most experiments were countable with this procedure. Foci were only difficult to count in the control experiment shown in Figure S6A, in which apparent foci numbers are indicated. BrdU staining was performed in cells treated with 10 μM BrdU for 24 hr. The cells were washed with PBS and pre-extracted (25 mM HEPES, pH 7.4, 50 mM NaCl, 1 mM EDTA, 3 mM $MgCl_2$, 300 mM sucrose, and 0.5% Triton X-100) for 20 min on ice before fixation with 4% paraformaldehyde and immunostaining. BrdU was detected by immunofluorescence under native conditions using a confocal laser microscope (Zeiss LSM880). EdU (Invitrogen) was used in some experiments. Survival rates were determined by visualizing cells with DAPI staining and counting the number of viable cells 1 week after γ-ray irradiation. Time-lapse imaging was performed on an

IncuCyte zoom system (Sartorius). Images were acquired once per hour, and cellular migration was assessed using a manual tracking tool in ImageJ. Migration velocity of irradiated cells was taken as the average of values measured 60–84 hr after irradiation, whereas the velocity of non-irradiated controls was taken as the average of values measured at 10 hr.

Antibodies. Antibodies against the following proteins and nucleotides were obtained from the indicated suppliers: γH2AX (9718, Cell Signaling Technology), 53BP1 (PC712-100ULCN, Merck), p21 (EAB-32448, Elabscience), p53 (D2H90, Cell Signaling Technology), β-actin (AC-74, Sigma), cGAS (D1D3G, Cell Signaling Technology), and BrdU (66241-1, ProteinTech).

Whole-genome sequencing analyses

DNA libraries were prepared using the TruSeq DNA PCR-free Library Preparation Kit (Illumina), following Illumina's recommended protocol. Whole-genome sequencing was performed on the NovaSeq 6000 at 2 × 150 bp read length. Mutation calling was performed using the Genomon2 pipeline (https://genomon.readthedocs.io/ja/latest/). In brief, sequencing reads were aligned to the mouse genome reference (mm10) using Burrows–Wheeler Aligner, version 0.7.10, with default parameter settings. PCR duplicates were eliminated using Picard-tools version 1.39 (http://picard.sourceforge.net/). Somatic mutations were detected by eliminating polymorphisms and sequencing errors. To achieve this, Genomon2 first discards any low-quality, unreliable reads and variants, which are defined by the following criteria: (i) mapping quality < 30; (ii) base call quality < 15; and (iii) reads with ≥ 5 SNVs or

indels, as performed in a previous study (Yokoyama et al., 2019). After further excluding variants that are not supported by a sufficient number of reads (variant reads ≥ 3) in tumor samples, variant allele frequencies (VAFs) ≥ 0.1 (for tumor sample) and ≤ 0.1 (for control sample). Putative germline variants are also excluded by comparing VAFs with matched control using the Fisher's test ($\leq 10^{-2}$), which also eliminates remaining additional sequencing errors. Detection of structural variations was performed by Genomon-SV (https://github.com/Genomon-Project/Genomon-SV). Briefly, Genomon-SV uses information from chimeric reads (containing breakpoints) and discordant read pairs. For each candidate structural variation, it realigns reads to the assembled contig sequence containing the structural variation breakpoint (variant sequence). Fisher's exact test was performed to compare the proportion of read pairs aligned to variant sequences relative to reference sequences in tumor versus matched control samples.

Mutational signature analyses, circus plot analyses, and correlation analyses of SV and SNV

Mutational signatures were analyzed using the Wellcome Trust Sanger Institute Mutational Signature Framework (http://jp.mathworks.com/matlabcentral/fileexchange/38724-wtsi-mutational-signature-framework). The optimal number of signatures was determined in accordance with the signature stabilities and average Frobenius reconstruction errors.

The circular representations of genomic alterations (SVs and SNVs) were visualized with BioCircos (Cui et al., 2016). SV and SNV rates were estimated in every 10 Mb (or 50 Mb) with moving averages; moving averages of 5 Mb (or 25 Mb) were

determined after counting the number of alterations every 1 Mb. To assess the correlation of SVs with SNVs, each point was plotted, and Pearson's correlation coefficients were calculated. Student's *t*-test was used for statistical analysis.

Supplemental References

Cui, Y., Chen, X., Luo, H., Fan, Z., Luo, J., He, S., Yue, H., Zhang, P., and Chen, R. (2016). BioCircos.js: an interactive Circos JavaScript library for biological data visualization on web applications. Bioinformatics *32*, 1740–1742.

Matsuno, Y., Atsumi, Y., Shimizu, A., Katayama, K., Fujimori, H., Hyodo, M., Minakawa, Y., Nakatsu, Y., Kaneko, S., Hamamoto, R., et al. (2019). Replication stress triggers microsatellite destabilization and hypermutation leading to clonal expansion in vitro. Nat. Commun. *10*, 3925.

Yokoyama, A., Kakiuchi, N., Yoshizato, T., Nannya, Y., Suzuki, H., Takeuchi, Y., Shiozawa, Y., Sato, Y., Aoki, K., Kim, S.K., et al. (2019). Age-related remodelling of oesophageal epithelia by mutated cancer drivers. Nature *565*, 312–317.
